# Photosynthetic Biomanufacturing in Mechanically Robust, 3D Printed Hydrogels

**DOI:** 10.1021/acssynbio.5c00366

**Published:** 2025-10-29

**Authors:** Jayce E. Taylor, Kinsey Drake, Nhu Tong, Jada A. Bezue, Alshakim Nelson, Shota Atsumi

**Affiliations:** † Department of Chemistry, 8789 University of California, Davis, Davis, California 95616, United States; ‡ Department of Chemistry, 7284 University of Washington, Seattle, Washington 98195, United States

**Keywords:** engineered living systems, metabolic engineering, biomaterials, cyanobacteria, engineered living materials

## Abstract

Engineered living materials (ELMs) integrate synthetic polymers with engineered cells to create systems that sense, respond, and adapt to their environment. While promising as sustainable alternatives to traditional materials, ELMs remain underexplored for use with photoautotrophic organisms. In this study, we evaluate the viability of the cyanobacterium *Synechococcus elongatus* PCC 7942, which converts carbon dioxide into valuable chemicals using light energy, in three hydrogel matrices previously shown to support heterotrophic cells. *S. elongatus* remained viable and metabolically active only in a hydrogel formed from bovine serum albumin-conjugated acrylates. When engineered to produce 2,3-butanediol (23BDO), encapsulated cells generated 719 mg L^–1^ over four days. Incorporating cells increased the compressive modulus of the material, while accumulated 23BDO reduced it, indicating that bioproduction influences mechanical properties. Fluorescence imaging confirmed high viability and physical immobilization. These results establish that cyanobacteria-based ELMs can enable autotrophic chemical production while modulating material mechanics for sustainable applications.

## Graphical abstract

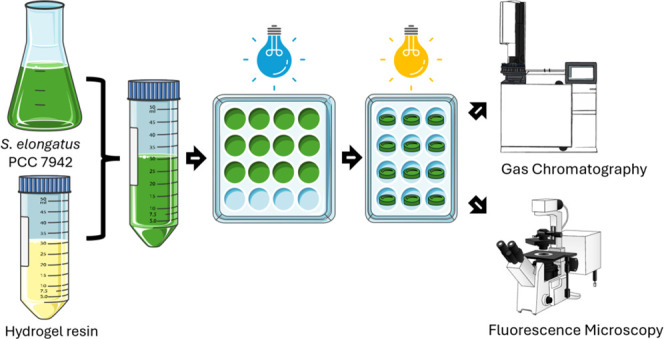

## Introduction

Our world is full of remarkable living materials—wood, coral, lichen, bone, and biofilms—that sense and respond to stimuli, self-repair, and self-organize.^1^ These materials typically consist of cells embedded in an extracellular matrix, which provides both structural support and protection.^2^ The cells allow dynamic responses to environmental cues, such as nutrient and water redistribution in trees,^3,4^ while matrices like biofilms shield bacteria from stress.^5^

Drawing inspiration from the dynamic and regenerative properties of natural living materials, engineered living materials (ELMs) are a convergence of materials science and synthetic biology that integrate the durability of abiotic substrates with the adaptability of biological systems, aiming to create biomimetic materials capable of sensing, responding, and evolving with their environment.^1,2,6–8^ ELMs are made by encapsulating engineered living cells within an extra-cellular matrix, which provides protection for the cells and dictates shape, flexibility, and durability.^1,2,6–8^ Engineered cells impart customizable, dynamic characteristics into the material, such as the ability to produce energy and chemical commodities, repair or degrade their extra-cellular matrix, perform bioremediation, and monitor or treat disease.^1,2,6–8^ ELMs are being explored for applications in healthcare, bioproduction, bioelectronics, bioremediation, and our built environment.^1,2,6–8^ While the applications and implications of ELMs are vast, it is still an emerging field in which foundational research is necessary. One such foundation is the compatibility between organisms and polymer matrices. Model organisms such as *Escherichia coli* are the main focus of investigation, but photosynthetic microorganisms have also garnered interest as ELM candidates due to their ability to sustain life and produce chemical commodities using sunlight and CO_2_.^9^ Studies have explored the encapsulation of photosynthetic cyanobacteria within polymer matrices such as sodium alginate,^10–12^ gelatin and sand,^13^ alginate-methylcellulose and sand,^14^ and porous ceramic.^15^ In addition to their self-sustaining metabolism, which makes cyanobacteria uniquely suited for scalable, carbon-negative ELMs, several synthetic biology tools have been developed for engineering cyanobacteria to generate value-added bioproducts.^10,16–18^

In this study, we build upon the growing foundation of ELM knowledge by evaluating *Synechococcus elongatus* PCC 7942 (hereafter *S. elongatus*) within three hydrogel matrices: polyethylene glycol diacrylate (PEGDA), bovine serum albumin-conjugated PEGDA (BSA-PEGDA), and F127-bis-urethane methacrylate (F127-BUM). While untested alongside cyanobacteria, PEGDA,^19^ BSA-PEGDA,^20–22^ and F127-BUM^23^ have proven biocompatible with other microorganisms. Furthermore, they have potential uses as modular, 3D-printable bioplastics with tunable mechanical properties.^19–21,23,24^ To assess functional performance and bioproduction capability, we encapsulated a strain of *S. elongatus,* strain AL3383 (see Materials and Methods), engineered to produce 2,3-butanediol (23BDO), a versatile chemical commodity and potential modulator of hydrogel mechanical properties.^25^ This work advances the foundational knowledge of ELMs by adding to the growing compendium of compatible organisms and polymer matrices and expanding on a potential method of bioproduction using photosynthesis.

## Materials and Methods

### Reagents

(*R*,*R*)-23BDO, PEGDA, glycerol, and lithium phenyl-2,4,6-trimethylbenzoylphosphinate (LAP) were purchased from Sigma-Aldrich. d-glucose, cycloheximide, and isopropyl-β-D-1-thiogalactopyranoside (IPTG) were purchased from Fisher Scientific. Spectinomycin was purchased from Research Products International. Kanamycin was purchased from VWR Life Sciences. Gentamycin was purchased from BioPlus Chemicals. Bovine serum albumin (BSA) was purchased from Bovogen Biologicals. F127-BUM was synthesized as previously described.^26^

### *S. elongatus* Strains

The wild-type strain was obtained from Prof. Susan Golden (UCSD). Strain AL3383 is genetically identical to strain AL2935 described in Kanno et al. (2017)^25^ and maintained as a new permanent stock. The genotype of AL3383 is NSI: *lacI* ^q^, *P*_trc_: *galP* (*E. coli*)-*zwf* (*E. coli*)-*gnd* (*E. coli*), NSIII: *lacI* ^*q*^, *P*_trc_: *alsD* (*Aeromonas hydrophila*)*-alsS* (*Bacillus subtilis*)*-adh* (*Clostridium beijerinckii*), *cp1*2: *P*_trc_: *prk* (*Synechococcus* sp. PCC 7002)-*rbcLXS* (*Synechococcus* sp. PCC 7002).

### Cultivation of *S. elongatus*

BG-11 medium was used to culture *S elongatus*.^27^ All BG-11 medium components were purchased from Sigma-Aldrich. The medium was prepared according to the standard composition, containing (per liter): 1.5 g NaNO_3_, 0.04 g K_2_HPO_4_, 0.075 g MgSO_4_·7H_2_O, 0.036 g CaCl_2_·2H_2_O, 0.006 g citric acid, 0.006 g ferric ammonium citrate, and 0.001 g Na_2_EDTA. The medium was supplemented with 1 mL of A5 metal mixture per liter, consisting of (per liter of stock): 2.86 g H_3_BO_3_, 1.81 g MnCl_2_·4H_2_O, 0.222 g ZnSO_4_·7H_2_O, 0.39 g Na_2_MoO_4_·2H_2_O, 0.079 g CuSO_4_·5H_2_O, and 0.049 g Co­(NO_3_)_2_·6H_2_O. The pH of the medium was adjusted to 7.0 and the medium was sterilized by filtration before use.

For experiments assessing cyanobacteria survival, *S. elongatus* was transferred to 10 mL of 1× BG-11 medium supplemented with 50 mM NaHCO_3_ and 50 μg mL^–1^ cycloheximide in a 10 mL glass tube. Cultures were grown at 30 °C with 100 rpm shaking under consistent light conditions (55 μE·s^–1^·m^–2^). Cultures were either monitored for pH change and adjusted back to pH–7 using 3.6 M HCl or centrifuged at 3200*g* for 10 min and resuspended in fresh 1× BG-11 medium with 50 mM NaHCO_3_ and 50 μg mL^–1^ cycloheximide. Once the culture established a vibrant green color, cells were transferred to 50 mL of fresh 1× BG-11 medium supplemented with 50 mM NaHCO_3_ and 50 μg mL^–1^ cycloheximide in 250 mL baffled glass flasks with a maximum circumference of 83 cm. Cultures were grown for 1 week with the same alternating pH adjustments and medium replacement as described above.

For experiments assessing 23BDO production, AL3383 was cultured in 50 mL of 1× BG-11 medium supplemented with 50 mM NaHCO_3_, 50 μg mL^–1^ cycloheximide, 20 μg mL^–1^ spectinomycin, 10 μg mL^–1^ kanamycin, and 10 μg mL^–1^ gentamycin in 250 mL baffled glass flasks. Cultures were grown for 1 week at 30 °C with 100 rpm shaking under consistent light conditions (55 μE·s^–1^·m^–2^) in a CO_2_ incubator with 2% atmospheric CO_2_. Cultures were either monitored for pH change and adjusted back to pH–7 using 3.6 M HCl, or centrifuged at 3200*g* for 10 min and resuspended in fresh 1× BG-11 medium with 50 mM NaHCO_3_, 50 μg mL^–1^ cycloheximide, 20 μg mL^–1^ spectinomycin, 10 μg mL^–1^ kanamycin, and 10 μg mL^–1^ gentamycin.

### Preparation of PEGDA Resin

PEGDA resin was prepared using 20 wt % PEGDA, 10 wt % glycerol, 1 wt % LAP, and 44 wt % 1× BG-11 medium. To prepare the resin, PEGDA was added to 1× BG-11 medium and vortexed until dissolved. Next, glycerol and LAP were added to the solution and vortexed to dissolve. PEGDA resin was wrapped in tinfoil to prevent light penetration and stored at 4 °C overnight.

### Preparation of BSA-PEGDA Resin

BSA-PEGDA resin was prepared using 30 wt % BSA, 10 wt % PEGDA, 1 wt % LAP, and 59 wt % 1× BG-11 medium. To prepare the resin, PEGDA was added to 1× BG-11 medium and vortexed until dissolved. Next, BSA was added in small aliquots and vortexed until dissolved after each addition. LAP was added to the solution and vortexed to dissolve. BSA-PEGDA resin was wrapped in tinfoil to prevent light penetration and stored at 4 °C overnight.

### Preparation of F127-BUM Resin

F127-BUM resin was prepared using 30 wt % F127-BUM, 1 wt % LAP, and 69 wt % 1× BG-11 medium. For easier handling, solution was kept on ice to prevent solidification. To prepare the resin, F127-BUM was added to 1× BG-11 medium and vortexed until dissolved. Next, LAP was added to the solution and vortexed to dissolve. F127-BUM resin was wrapped in tinfoil to prevent light penetration and stored at 4 °C overnight.

### Constructing an ELM “Curing Oven”

A cardboard box approximately 28.5 cm by 21 cm by 9.5 cm was lined with aluminum foil. On one face of the box (28.5 cm by 21 cm), a hole approximately 13 cm by 8 cm was cut. This hole served as an opening to the “oven” to insert the silicon mold or UV lamp. After placing the silicon mold into the oven, a 395–400 nm UV lamp (15.5 cm × 14 cm × 3 cm, obtained from Amazon) was positioned laying over the hole, facing into the box.

### Constructing ELM Pucks

*S. elongatus* cultures were centrifuged at 3200*g* for 10 min and the supernatant discarded. Cells were then resuspended in 1× or 5× BG-11 medium and adjusted to OD_730_ = 20 or 100. OD_730_ was measured using a Microtek Synergy H1 plate reader (BioTek). For survival assays, *S. elongatus* was combined with resin in a 1:20 ratio of cells to resin, with a final OD_730_ = 1. For 23BDO production assays, AL3383 was combined with resin in a 1:20 ratio of cells to resin, with a final OD_730_ = 5. Cell-resin mixture was then pipetted into silicon molds with a volume of 300 μL, forming ELM “pucks”. The mold was placed in a constructed “curing oven” and cured under 395–400 nm UV light for 1 h.

### Survival Assays

After curing for 1 h under 395–400 nm UV light, ELM pucks were removed from the silicon mold and placed in 3 mL of medium in a covered 12-well culture plate. Assay medium consisted of 1× BG-11 medium, 20 mM NaHCO_3_, 50 μg mL^–1^ cycloheximide. Plates containing ELM pucks in medium were cultured for 2 days at 30 °C with 100 rpm shaking under consistent light conditions (55 μE·s^–1^·m^–2^).

### Mechanical Characterization of ELMs

After culturing, ELM pucks (*x* = 10 mm, *y* = 10 mm, *z* = 5 mm) were washed twice with culture media and dried at ambient conditions for 3 days (bioplastic state). Pucks were rehydrated overnight in 5× BG-11 medium at ambient temperature. A Newton Test Machine electromechanical test frame with 1 kN load cell and a crosshead rate of 1 mm/min was used to perform compression experiments on the pucks. For samples in the bioplastic state, an Instron Test Machine with a 50 kN load cell and a crosshead rate of 1 mm/min was used to assess the compressive modulus. The compressive modulus was calculated based on the slope of the stress (kPa)-strain (mm/mm) curve in the linear region of the test. Toughness was calculated as the area under the stress–strain curve prior to mechanical failure.

### Degree of Swelling and Media Uptake

The degree of swelling (q) was determined by monitoring the volume change of dried pucks (bioplastic state) after overnight incubation in 5× BG-11 medium (rehydrated state). Pucks in the bioplastic state were incubated in 5× BG-11 medium overnight at room temperature, and the change in volume of dehydrated and rehydrated states was calculated using measurements made with a digital caliper. The degree of swelling was calculated using the following equation.

1 $$q=\frac{\left(Rehydrated\;volume-dried\;volume\right)}{Dried\;volume}$$

Medium uptake (MU) capacity (%) was calculated by the change of dehydrated and rehydrated states using the following equation.

2 $$MU\left(\%\right)=\frac{\left(Rehydrated\;mass-dried\;mass\right)}{\left(Rehydrated\;mass\right)}\times 100$$

### Thermogravimetric Analysis of ELMs

Eight mg samples of ELM pucks in the bioplastic state were placed in aluminum pans and heated on a TA Instruments Q5000 IR thermogravimetric analyzer (TGA). Samples were heated from 10 to 600 °C at a rate of 10 °C/min with isothermal holds at 100 °C for 30 min and 235 °C for 60 min to allow for complete removal of water and 23BDO, respectively.

### Photomixotrophic and Photoautotrophic Production of 23BDO

After curing for 1 h under 395–400 nm UV light, ELM pucks were removed from the silicon mold and placed in 3 mL of production medium in a covered 12-well culture plate. Production medium consisted of the appropriate concentration of BG-11 medium, the appropriate concentration of NaHCO_3_, the appropriate concentration of glucose, 10 mg L^–1^ thiamine, 50 μg mL^–1^ cycloheximide, 20 μg mL^–1^ spectinomycin, 10 μg mL^–1^ kanamycin, 10 μg mL^–1^ gentamycin, and 1 mM IPTG. Plates containing pucks in production medium were cultured for 4 days at 30 °C with 100 rpm shaking under consistent light conditions (55 μE·s^–1^·m^–2^). After 4 days, medium evaporation from each well was determined via weighing by difference. One mL samples of medium were collected from each well for analysis.

### Quantification of 23BDO

Quantification of 23BDO followed a previously established method.^28^ Culture supernatant was analyzed using gas chromatography (GC) (Shimadzu) equipped with a flame-ionization detector and a DB-WAX column (30 m, 0.32 mm internal diameter, 0.5 μm film thickness, Agilent Technologies). Ultrahigh purity helium was used as the carrier gas. For each run, GC oven temperature was held at 105 °C for 1 min, increased with a gradient of 10 °C min^–1^ until 200 °C and held for 2 min. Inlet temperature was set at 200 °C, while detector temperature was set to 330 °C.

Samples were prepared by centrifuging at 17,000 g for 2 min to concentrate potential debris. Aliquots of supernatant were extracted from the top of each sample and combined in equal volumes with an internal standard of 500 mg L^–1^ 1,3-propanediol (1,3-PD) in 1× BG-11 medium.

The measured concentration of 23BDO in each sample was adjusted to account for medium evaporation over the course of the experiment.

### Fluorescence Imaging

Fluorescence imaging of AL3383 cells embedded in BSA-PEGDA ELM pucks was performed following a protocol adapted from Datta et al.^10^ SYTOX Blue (5 μM; Thermo Fisher) was used to stain sacrificial 0 h ELM pucks, incubated in the dark for 30 min in 1× BG-11 medium to prior to imaging. Remaining ELMs were cultured for 4 days as described in photomixotrophic and photoautotrophic production of 23BDO before staining.

Imaging was conducted on a Zeiss 980 Laser Scanning Microscope with Airyscan 2. SYTOX Blue was detected using 405 nm excitation and 450–510 nm emission; chlorophyll autofluorescence was detected using 488 nm excitation and 685–720 nm emission. Z-stacks were acquired for 3D image reconstruction.

## Results and Discussion

### Assessing Hydrogel Compatibility with *S. elongatus*

Initial trials for encapsulating *S. elongatus* were performed using a PEGDA hydrogel matrix. PEGDA was chosen for initial encapsulation assessment due to its history of biocompatibility with tissues, *E. coli*, and *Saccharomyces cerevisiae*.^24,29–31^ Previous work showed that *S. elongatus* remained viable when first encapsulated in sodium alginate micro beads, followed by secondary encapsulation in PEGDA.^12^ Hydrogel samples containing wildtype *S. elongatus* (hereafter “pucks”) were incubated in BG-11 medium for 24 h. During this period, the pucks changed from a vibrant green to nearly transparent, indicating chlorosis, the loss of chlorophyll and a sign of cell death (Figure 1A).^32,33^

**Figure 1:**
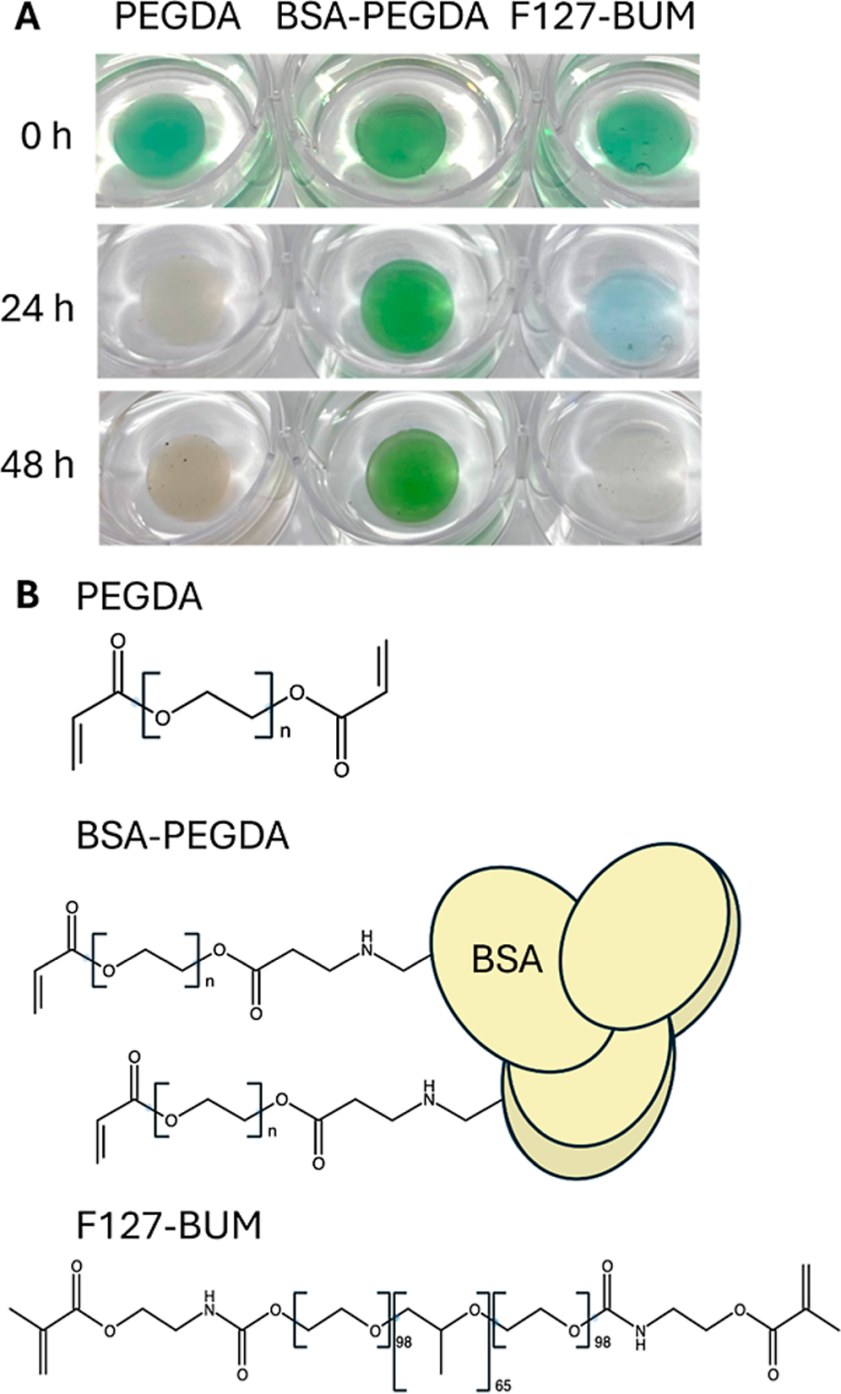
Survival of *S. elongatus* in Different ELM Hydrogel Matrices. (A) Viability of *S. elongatus* encapsulated in hydrogels composed of PEGDA, BSA-PEGDA, and F127-BUM. (B) Chemical structures of PEGDA, BSA-PEGDA, and F127-BUM. Cross-linking occurs via the acrylate groups of PEGDA and BSA-PEGDA or the methacrylate groups of F127-BUM through free radical polymerization.

To rule out UV light as the cause of cell death, aliquots of *S. elongatus* in BG-11 medium were irradiated for 0, 30, and 90 min, and then cultured in BG-11 medium for 72 h. All cultures continued to grow, becoming denser over time, although the culture that received 90 min of UV light grew at a slower rate (Figure S1A). To assess the toxicity of resin components, *S. elongatus* cells were cultured in BG-11 medium containing PEGDA, the photoinitiator LAP, or both. All conditions containing PEGDA and/or LAP caused chlorosis, indicating toxicity (Figure S1B).

After confirming the toxicity of PEGDA-based hydrogels on *S. elongatus*, two additional polymers, BSA-PEGDA and F127-BUM, were tested. BSA-PEGDA has demonstrated biocompatibility with *E. coli* and *S. cerevisiae* and can be 3D-printed into mechanically functional components for load-bearing applications.^20,21,34^ F127-BUM has demonstrated biocompatibility with *E. coli* and yeasts, can be used to 3D print structures, and has been shown to protect cells through lyophilization, storage, and rehydration.^23,35–37^

Like PEGDA, *S. elongatus* encapsulated in F127-BUM demonstrated chlorosis, but unlike PEGDA, *S. elongatus* encapsulated in BSA-PEGDA remained vibrant green past 48 h (Figure 1A). The polymerization of PEGDA, BSA-PEGDA, and F127-BUM all involve free radical initiated cross-linking, where the photoinitiator LAP is activated by UV light, inducing a chain reaction cross-linking acyl group (PEGDA and BSA-PEGDA) or methacrylate groups (F127-BUM) (Figure 1B). These residues can also become radicalized using UV light without the use of a photoinitiator, although at lower rates.^38^ In the case of BSA-PEGDA, PEGDA also reacts with the lysine residues of BSA through an aza-Micheal addition.^20^ We theorize that the inclusion of bulky BSA proteins within the polymer matrix provides either a physical buffer between cells and radicalized PEGDA molecules, or acts as a quencher of excess free radicals, either from PEGDA or the photoinitiator. Neither PEGDA nor F127-BUM contain this bulky protein, which may allow for the polymerization process to come in direct contact with *S. elongatus* cells. In another study, efforts to encapsulate the cyanobacteria *Synechococcus* sp. PCC 7002 in F127-BUM have been successful only with low polymer concentrations that yield mechanically soft ELMs after UV polymerization.^39^ Given the observed biocompatibility between *S. elongatus* and BSA-PEGDA, a hydrogel solution composed of BSA-PEGDA, LAP, and BG-11 medium was used for further bioproduction analysis.

### Mechanical Characterization of BSA-PEGDA ELMs Containing *S. elongatus* and 23BDO

To evaluate the structural integrity of *S. elongatus*-laden BSA-PEGDA pucks containing 23BDO, the target bioproduct of this study, uniaxial compression tests were performed on BSA-PEGDA ELM pucks with or without *S. elongatus* (OD_730_–5) and varying levels of 23BDO. The compressive modulus was determined from the linear region of the stress–strain curve (Figure S2A). Incorporation of *S. elongatus* into the BSA-PEGDA matrix significantly increased the compressive modulus compared to acellular hydrogels (*p* < 0.001), indicating that the embedded cells contributed to enhanced mechanical strength (Figure 2A). In contrast, inclusion of 23BDO at concentrations of 760 mg/L and 1350 mg/L led to a reduction in compressive modulus, suggesting that the hydroxyl groups on 23BDO act as a plasticizer similar to glycerol (Figure 2A).^19,20^ Notably, thermogravimetric analysis of the ELM pucks in the Bioplastic state showed similar water concentrations across all conditions, indicating that mechanical softening is due to concentration-dependent effects of 23BDO rather than water content (Figure 2B).

**Figure 2:**
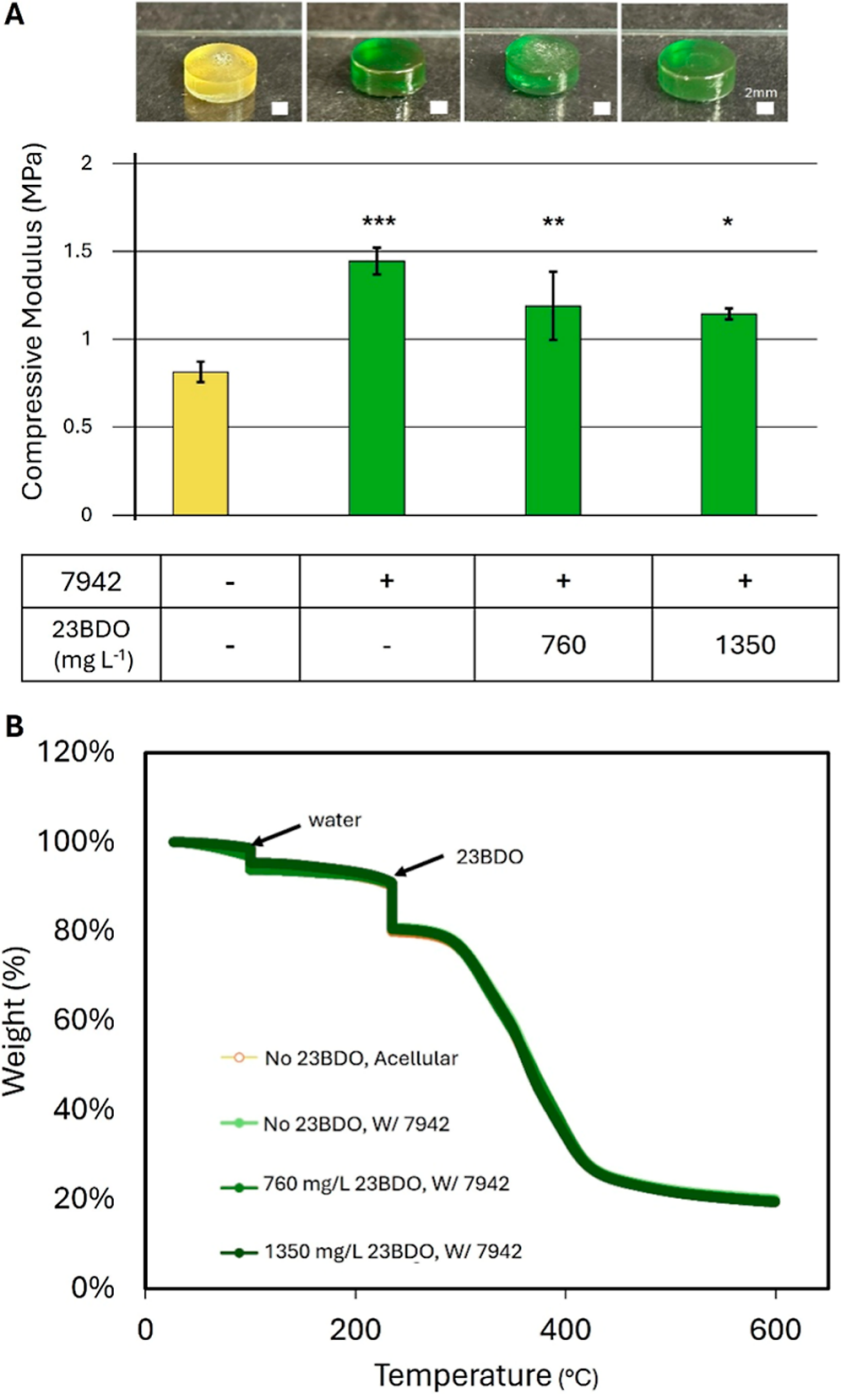
Mechanical Properties and Thermal Behavior of Cyanobacteria-Laden BSA-PEGDA ELMs. (A) Uniaxial compression testing of BSA-PEGDA pucks containing AL3383, with or without 760 mg/L or 1350 mg/L 23BDO. Acellular BSA-PEGDA samples without 23BDO served as controls. Statistical analysis was performed using one-way ANOVA followed by Dunnett’s posthoc test (two-sided), comparing each group to the acellular control (*n* = 3 per group; ns: not significant; *P < 0.05; **P < 0.01; ***P < 0.001). Data are presented as mean ± SD B. Thermogravimetric analysis of BSA-PEGDA ELMs in the Bioplastic state, containing *S. elongatus* with or without 760 mg/L or 1350 mg/L 23BDO. Acellular BSA-PEGDA samples without 23BDO were used as controls.

To investigate the effect of *S. elongatus* and 23BDO on the hydration behavior of BSA-PEGDA ELM pucks, we measured medium uptake capacity. The presence of *S. elongatus* and the addition of 23BDO at concentrations of 760 mg/L and 1350 mg/L did not significantly affect the medium uptake capacity or swelling of the hydrogels (Figure S2B,C). These results indicate that neither cell encapsulation nor 23BDO production compromises the material’s ability to maintain shape fidelity and retain medium.

To evaluate the toughness of BSA-PEGDA ELM pucks containing *S. elongatus* and 23BDO, we used the same sample combinations as in the above experiments, including pucks with or without *S. elongatus* and with 760 mg/L or 1350 mg/L 23BDO. Toughness showed no significant differences across conditions (Figure S2D), reducing the risk of premature obsolescence of the ELM due to fracture during bioproduction cycles. These mechanically robust formulations stand apart from the softer biopolymer systems typical of cyanobacteria ELMs, providing new opportunities for functional ELMs.

### Photomixotrophic Production of 23BD by *S. elongatus* in a BSA-PEGDA ELM

To assess the bioproduction capacity of *S. elongatus* encapsulated within a BSA-PEGDA hydrogel puck, we used AL3383 (see Materials and Methods), a previously established *S. elongatus* strain capable of producing 23BDO.^25^ 23BDO is a precursor to fuel additives, polymers, and industrial solvents, and is not reassimilated or consumed by *S. elongatus*.^40^ Additionally, hydrogen bonding of 23BDO to water could potentially be used to increase hydrogel hydration, a challenge for ELMs in arid climates.^19^

Strain AL3383 contains a genome-integrated pathway that converts pyruvate to 23BDO via acetolactate synthase (ALS, *B. subtilis*), acetolactate decarboxylase (ALDC, *A. hydrophila*), and secondary alcohol dehydrogenase (sADH, *C. beijerinckii*) (Figure 3A).^25^ Additionally, AL3383 possesses an inducible glucose consumption pathway, which can enhance both 23BDO production and CO_2_ fixation.^25^ The glucose import pathway consists of a galactose proton symporter (GalP, *E. coli*), glucose 6-phosphate dehydrogenase (Zwf, *E. coli*), 6-phosphogluconate (6PG) dehydrogenase (Gnd, *E. coli*).

**Figure 3:**
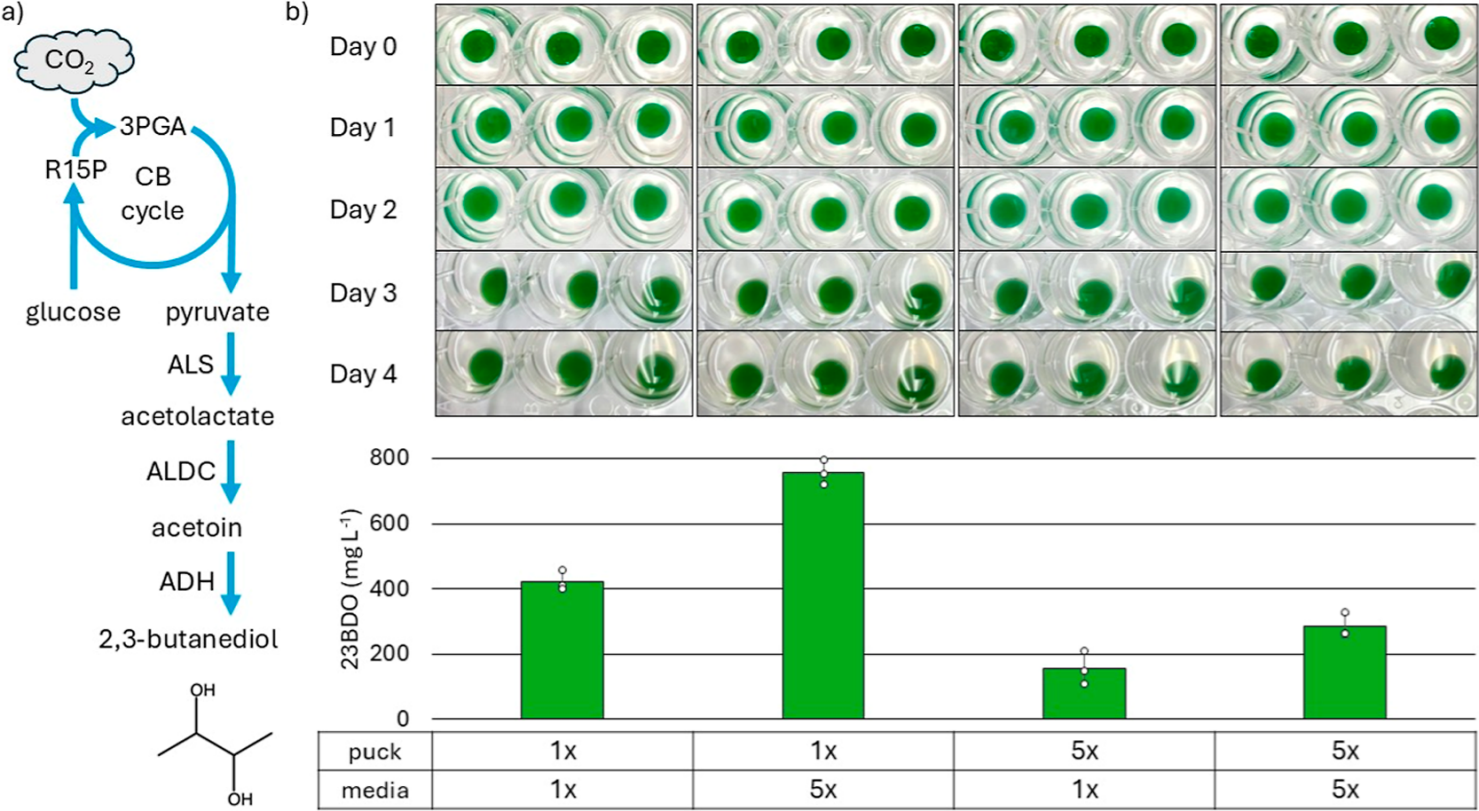
Photomixotrophic Production of 23BDO by *S. elongatus* encapsulated in ELMs. (A) Engineered 23BDO biosynthetic pathway in AL3383. CB cycle, Calvin–Benson cycle; 3PGA, 3-phosphoglycerate; R15P, ribulose-1,5-bisphosphate; ALS, acetolactate synthase; ALDC, acetolactate decarboxylase; ADH, alcohol dehydrogenase. B. 23BDO production from CO_2_ and glucose after 4 days in ELM pucks incubated at 30 °C under continuous light in either 1× or 5× BG-11 medium, using pucks formulated with 1× or 5× BG-11 medium. Experiment was performed using biological replicates (*n* = 3). Error bars represent standard deviation. A one-way ANOVA indicated statistically significant differences among groups (F­(3, 8) = 130.7, *p* = 3.91 × 10^–7^). Posthoc Tukey’s HSD tests showed that mean 2,3-butanediol production differed significantly between the following pairs: [1×, 1×] vs [1×, 5×] (*p* = 2.89 × 10^–5^, 95% CI = 230.8–435.3); [1×, 1×] vs [5×, 1×] (*p* = 1.49 × 10^–4^, 95% CI = 164.1–368.6); [1×, 1×] vs [5×, 5×] (*p* = 0.0114, 95% CI = 34.6–239.1); [1×, 5×] vs [5×, 1×] (*p* = 2.91 × 10^–7^, 95% CI = 497.1–701.6); [1×, 5×] vs [5×, 5×] (*p* = 2.16 × 10^–6^, 95% CI = 367.7–572.2). The difference between [5×, 1×] and [5×, 5×] was not statistically significant (*p* = 0.155, 95% CI = 27.2–231.7).

To validate system performance and ensure a detectable output, ELM pucks containing AL3383 were initially tested under photomixotrophic conditions, using both CO_2_ and glucose as carbon sources to bolster 23BDO production. To test 23BDO production, AL3383 was first encapsulated in BSA-PEGDA. Both 1× and 5× BG-11 medium concentrations were used in the resin formulation and production medium to assess the effect of nutrient availability. Production medium also contained 20 mM NaHCO_3_ as a source of CO_2_ and 10 g L^–1^ glucose. ELM pucks were cultured for 4 days, during which medium samples were extracted on day 0 and day 4. After 4 days, ELMs containing 1× BG-11 medium within the puck and 5× BG-11 production medium had produced the most 23BDO with an average of 751 mg L^–1^ (Figure 3B). All other conditions also supported 23BDO production, with yields ranging from 155 to 420 mg L^–1^ depending on the combination of BG-11 concentrations in the puck and production medium. Further experiments used 1× BG-11 medium in the ELM pucks and 5× BG-11 production medium.

### Fluorescence Imaging of *S. elongatus* in a BSA-PEGDA ELM

To observe the spatial distribution and general physiological state of encapsulated *S. elongatus* in BSA-PEGDA, ELM pucks containing strain AL3383 were subjected to fluorescence microscopy imaging. SYTOX Blue, a membrane-impermeant nucleic acid stain, was used to visualize cells within the polymer matrix.^10^ Although the stain has been reported to penetrate only dead cells, we observed staining in nearly all cells. This may be due to altered membrane permeability during encapsulation or fixation and highlights the limitations of interpreting SYTOX Blue as a strict viability marker in this context. Therefore, we used the stain solely to enhance contrast and enable visualization of cells within the hydrogel.

To provide complementary information, we also imaged chlorophyll autofluorescence, which is present in photosynthetically active cells and can offer qualitative insight into the metabolic state. Chlorophyll fluorescence was collected at an excitation of 488 nm and emission of 685–720 nm (Figure S3A,D). The fluorescence of SYTOX Blue was collected at an excitation of 405 nm and emission of 450–510 nm (Figure S3C,F). These two fluorescence channels were overlaid to visualize the distribution of AL3383 cells (Figure S3B,E). While not intended as a viability assay, this dual-channel imaging qualitatively suggests that many encapsulated cells maintained chlorophyll fluorescence over 4 days of photomixotrophic 23BDO production. Live imaging under the microscope showed the cells to be physically immobilized by the polymer matrix, as indicated by their lack of movement compared to nonencapsulated cells. 3D Z-stack images and movies are available upon request.

### Photoautotrophic Production of 23BDO by *S. elongatus* in a BSA-PEGDA ELM

The prime motivation of using photosynthetic organisms within ELMs is their ability to produce energy and biomass using readily available resources: light and CO_2_. Therefore, in addition to photomixotrophic production, we also tested AL3383 ELMs for photoautotrophic 23BDO production using just CO_2_ as a carbon feedstock.

Using the best BG-11 medium conditions from photomixotrophic experimentation, 1× BG-11 medium in pucks and 5× BG-11 production medium, we analyzed the ELM’s ability to produce 23BDO when presented with 0, 10, 20, 50, and 100 mM NaHCO_3_ as a CO_2_ source. After 4 days, ELMs supplied with 20 mM NaHCO_3_ had produced the most 23BDO with an average of 719 mg L^–1^ (Figure 4). Increasing or decreasing the bicarbonate concentration led to reduced production, with yields ranging from 386 mg L^–1^ at 10 mM to 625 mg L^–1^ at 50 mM. ELM pucks with no NaHCO_3_ supplementation produced the least 23BDO, at 247 mg L^–1^.

**Figure 4:**
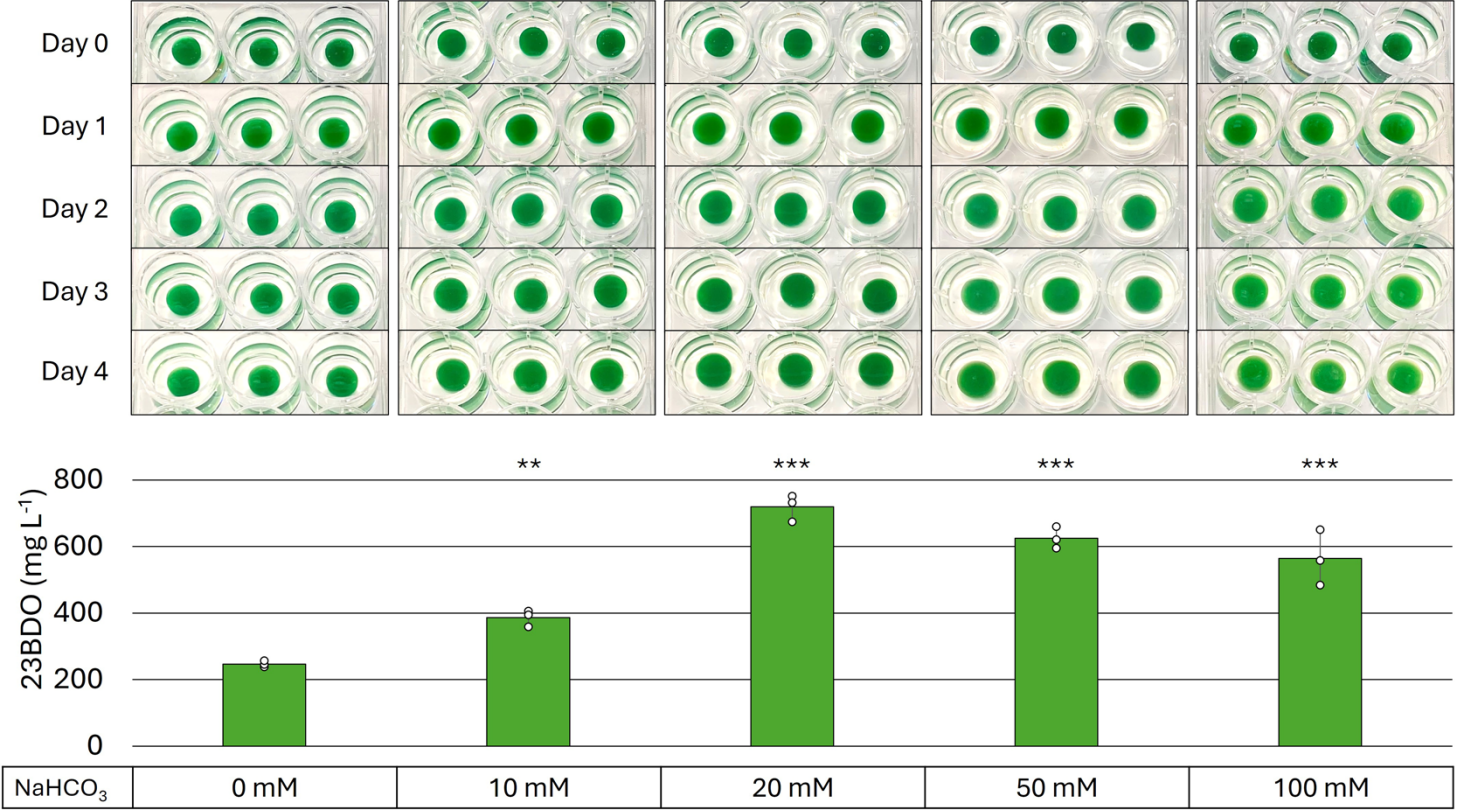
Photoautotrophic Production of 23BDO by *S. elongatus* encapsulated in ELMs. 23BDO production by AL3383 from CO_2_ after 4 days in BSA-PEGDA ELM pucks incubated at 30 °C under continuous light in 5× BG-11 medium. Pucks were formulated with 1× BG-11 medium and supplemented with varying concentrations of NaHCO_3_ (0, 10, 20, 50, and 100 mM) as the CO_2_ source. Experiment was performed using biological triplicates, *n* = 3. Statistical analysis was performed using one-way ANOVA followed by Dunnett’s post-hoc test (two-sided), comparing each group to 0 mM NaHCO_3_ as a control (ns: not significant; *P < 0.05; **P < 0.01; ***P < 0.001). Error bars represent standard deviation.

## Conclusion

In this study, we demonstrated that *S. elongatus* can be encapsulated in BSA-conjugated hydrogels to form photosynthetically active ELMs capable of producing 23BDO from light and CO_2_. In contrast, other hydrogel matrices such as PEGDA and F127-BUM proved toxic, highlighting the importance of material compatibility for *S. elongatus* survival. Our findings provide foundational knowledge for using *S. elongatus* in hydrogel-based ELMs, which offer modularity, biocompatibility, and potential for additive manufacturing, critical features for future applications in healthcare, bioproduction, bioelectronics, bioremediation, and our built environment.^1,2,6–8^ One promising future direction is the development of Engineered Living Systems (ELiS), where multiple microbial species are encapsulated, but remain physically separated, within an extracellular matrix.^41^ Spatial separation of species by a matrix that still allows for exchange of metabolites encourages the development of microbial consortia and division of labor, with cyanobacteria potentially acting as autotrophic primary producers that fix CO_2_ and supply metabolites to heterotrophic partners. This spatially organized coculture strategy could improve synthetic circuit performance, increase resilience, and reduce competition for feedstocks, paving the way toward mechanically robust, self-sustaining living materials for real-world environments.^2,41,42^

## Supplementary Material



## Data Availability

The data sets generated in this study are available from the corresponding author on reasonable request.

## Notes

Figure S1 Identifying components affecting the survival of *S. elongatus* in PEGDA-based ELM. Figure S2 Additional Mechanical and Physical Characterization of *S. elongatus*-Laden BSA-PEGDA ELMs. Figure. S3 Fluorescence imaging of *S. elongatus* in ELMs (PDF)
