# Supplementary material for: Photosynthetic Biomanufacturing in Mechanically Robust, 3D Printed Hydrogels
Source: ACS Synth Biol. 2025 Oct 29;14(11):4439–46. doi: 10.1021/acssynbio.5c00366 (PMC12645572; doi:10.1021/acssynbio.5c00366)
Supplement: Supplementary file 1 [file sb5c00366_si_001.pdf]

Supplementary Information

Photosynthetic Biomanufacturing in Hydrogels

Jayce E. Taylor[1,ǂ], Kinsey Drake[2,ǂ], Nhu Tong[1], Jada A. Bezue[1], Alshakim Nelson[2,*], and Shota Atsumi[1,*]

[1]Department of Chemistry, University of California, Davis, Davis, CA, 95616, USA

[2]Department of Chemistry, University of Washington, Seattle, WA, 98195, USA

ǂThese authors contributed equally

*To whom correspondence may be addressed: E-mail: alshakim@uw.edu, satsumi@ucdavis.edu

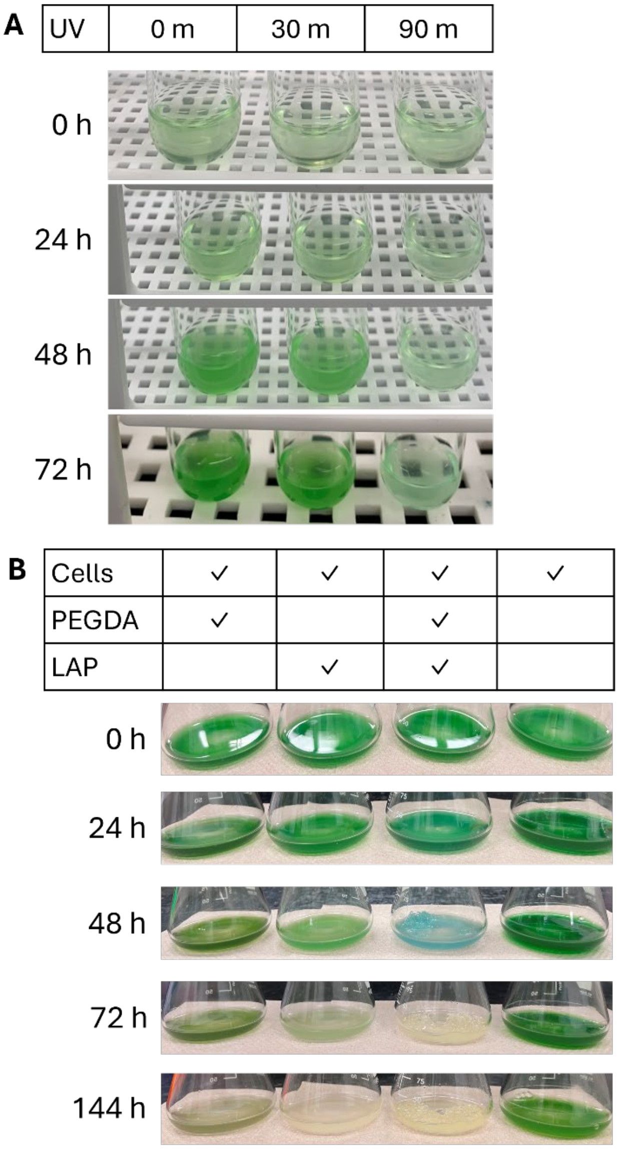

Fig. S1 Identifying components affecting the survival of *S. elongatus* in PEGDA-based ELM*.*
A. *S. elongatus* cells were exposed to 395–400 nm light for 0, 30, or 90 minutes, then cultured in BG-11 medium for 72 hours. **B.** *S. elongatus* cells were cultured for 144 hours in BG-11 medium containing 20% PEGDA, 10% LAP, or a combination of both to assess potential cytotoxicity.

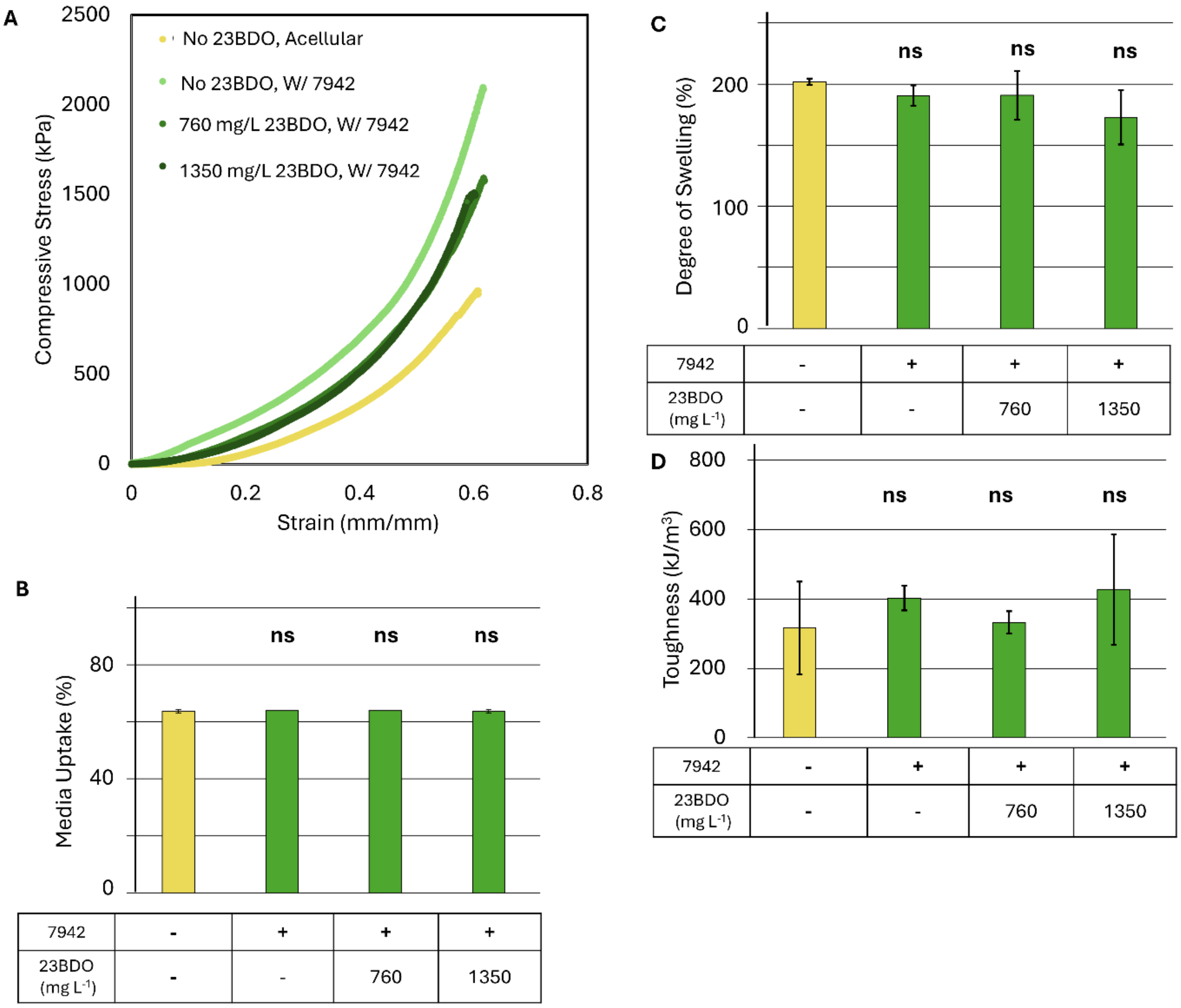

Fig. S2 Additional Mechanical and Physical Characterization of *S. elongatus* -Laden BSA-PEGDA ELMs. A. Representative raw stress–strain curves from uniaxial compression tests on BSA-PEGDA ELMs containing *S. elongatus* PCC7942 and 23BDO. **B-D.** Medium uptake, degree of swelling, and toughness of BSA-PEGDA pucks containing *S. elongatus* PCC7942 and either 760 mg/L or 1350 mg/L 23BDO. For **B ,C,** and **D**, statistical comparisons were performed using one-way analysis of variance (ANOVA) followed by Dunnett's multiple comparisons test (two-sided), comparing *S. elongatus*-containing samples to the acellular control (n = 3 per group; ns: not significant; *P < 0.05; **P < 0.01; ***P < 0.001). Data are presented as mean ± SD; n = 3 or more replicates.

©

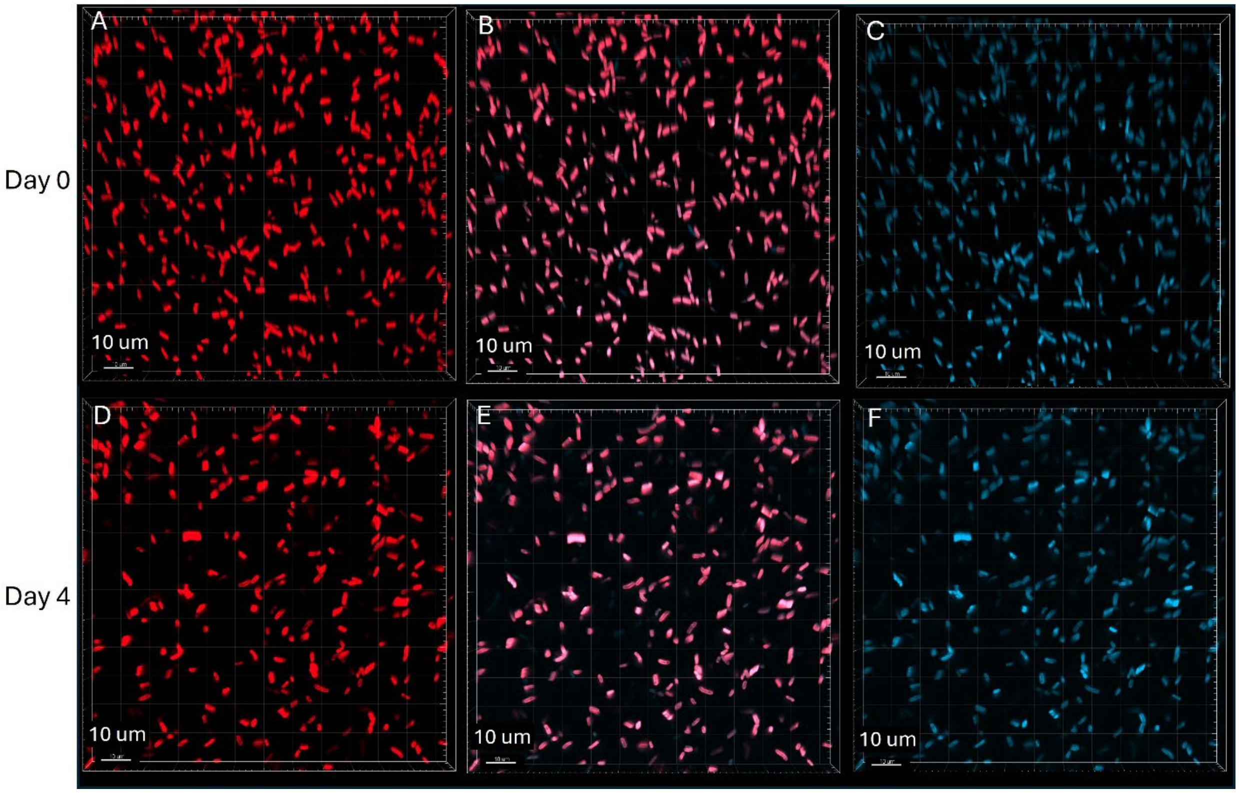

Fig. S3 Fluorescence imaging of *S. elongatus* in ELMs.

Fluorescence microscopy of strain AL3383 encapsulated in BSA-PEGDA hydrogels on Day 0 (**A–C**) and Day 4 (**D–F**) of the 23BDO photomixotrophic production experiment. **A, D:** Chlorophyll autofluorescence of encapsulated cells. **C, F:** SYTOX Blue staining of cells within the hydrogel matrix. **B, E:** Merged fluorescence images, where cells exhibiting both chlorophyll autofluorescence and SYTOX Blue staining appear pink, and cells with only SYTOX Blue staining appear blue.
